# Same Score, Different Risk: Toward Individualized Anticoagulation After Successful AF Ablation: A Narrative Review for the Post-Ablation Antithrombotic Decision in Atrial Fibrillation

**DOI:** 10.31083/RCM51896

**Published:** 2026-06-29

**Authors:** Moez Alnazeer, Jerry Fan, Peter Cheung, Javier Banchs, Gregory Olsovsky

**Affiliations:** ^1^Department of Internal Medicine, Division of Cardiology, Section of Cardiac Electrophysiology, Baylor College of Medicine, Temple, TX 76504, USA

**Keywords:** atrial fibrillation, catheter ablation, anticoagulant, thromboembolism, risk assessment

## Abstract

Current guidelines recommend that long-term oral anticoagulation (OAC) after atrial fibrillation (AF) ablation be guided by CHA_2_DS_2_-VASc rather than ablation success. However, two recent randomized trials, ALONE-AF and OCEAN, observed very low stroke and systemic embolism rates in rigorously selected post-ablation populations. Because events were rare and neither trial was powered for noninferiority for stroke, they do not definitively establish equivalence with continued OAC. Integrating these data with AF burden studies, atrial cardiomyopathy research, and post-ablation registries, we examine the hypothesis that a given CHA_2_DS_2_-VASc score may confer lower stroke risk when AF burden is durably suppressed. We identify five domains clinicians should weigh: time since ablation, monitoring quality, AF burden, atrial substrate, and baseline thromboembolic risk. Within patients with 1–2 non-sex CHA_2_DS_2_-VASc risk factors, arrhythmia-free beyond 12 months with robust monitoring, and without advanced atrial cardiomyopathy, the discussion of OAC de-escalation can now be informed by randomized data rather than extrapolation alone. We also identify the uncertainties that remain and the populations in whom guideline-directed OAC remains the standard of care.

## 1. Introduction: Same Score, New Denominator

A 65-year-old man with paroxysmal atrial fibrillation (AF) and diabetes returns 18 months after catheter ablation. An implantable loop recorder (ILR) has shown no recurrent AF, and his main concern is easy bruising on apixaban. His question is simple: “If the ablation worked, do I really still need this blood thinner?”

Contemporary AF guidelines largely answer “yes”. Both US and European documents recommend that long-term oral anticoagulation (OAC) be guided by estimated thromboembolic risk, typically assessed with CHA_2_DS_2_-VASc or CHA_2_DS_2_-VA and not by perceived ablation success [1,2]. In practice, however, many clinicians individualize beyond this framework, and two recent randomized trials–ALONE-AF and OCEAN–now provide data in precisely this space.

In this review, we synthesize these trials with real-world registry data, device-based AF-burden evidence, and the evolving concept of atrial cardiomyopathy to examine the emerging evidence and identify the key considerations that should inform individualized post-ablation antithrombotic decisions. We frame this explicitly as a hypothesis supported by recent trial data, not as a mandate to abandon guideline-directed care, and we delineate both the patients in whom de-escalation may be reasonable and those in whom it remains unsupported.

We searched PubMed and MEDLINE through February 2026 using terms including atrial fibrillation, catheter ablation, anticoagulation, stroke prevention, and AF burden. We prioritized randomized controlled trials, large registry studies, and consensus statements published in English. This is a narrative review; no formal systematic search protocol or study quality assessment was applied.

## 2. Guideline Logic Versus Real-World Practice

Stroke prevention in AF is anchored in a straightforward construct: estimate thromboembolic risk with CHA_2_DS_2_-VASc and offer long-term OAC when CHA_2_DS_2_-VASc–based risk is sufficiently high per guideline thresholds, regardless of rhythm control strategy [1,2]. These recommendations reflect the evidence base at the time of writing: observational data with inconsistent rhythm monitoring and concern that ablation might reduce symptoms without fully abolishing thromboembolic risk. Crucially, these guidelines were finalized before the publication of ALONE-AF and OCEAN, and the present review attempts to integrate these new data post-dating guideline finalization into clinical decision-making.

Real-world practice, however, is more heterogeneous. Large registry and claims analyses demonstrate that OAC discontinuation after ablation is common, often occurring within the first year, and not infrequently in patients with CHA_2_DS_2_-VASc ≥2 [3]. The result is a widening gap between guideline-based decision-making and clinical behavior. Clinicians confronting patients with no detectable AF on continuous monitoring struggle to justify indefinite anticoagulation in the face of bruising, falls, gastrointestinal bleeding, or treatment burden. ALONE-AF and OCEAN now provide randomized data that force us to revisit whether a score-only approach remains sufficient after successful ablation.

## 3. Post-Ablation OAC Discontinuation Trials: ALONE-AF and OCEAN

Two recent randomized trials directly address whether OAC can be discontinued after successful AF ablation, enrolling patients in a durable sinus rhythm ≥12 months after ablation with low-to-moderate CHA_2_DS_2_-VASc scores (≈2–3). Each tests a different step-down strategy; Table 1 (Ref. [4,5]) details design and outcomes. Below we highlight the interpretive essentials.

**Table 1.** **Key randomized trials informing post-ablation OAC discontinuation**.

Trial [Ref]	N	Population	Mean CHA_2_DS_2_-VASc	Strategies	Follow-up	Monitoring Intensity	Key Efficacy / Safety Results	Key Takeaway
ALONE-AF [4]	840	Post-ablation, ≥12 mo arrhythmia-free, ≥1 non-sex risk factor; South Korea	≈2	Stop DOAC (no OAC) vs continue DOAC	2 years (Final follow-up to June 2025)	ECG each visit + 24–72 h Holter ≥ every 6 mo; additional monitoring for symptoms	Primary composite (stroke/SE/major bleed): 0.3% vs 2.2% (absolute diff −1.9%; 95% CI −3.5 to −0.3; *p* = 0.02). Ischemic stroke: 0.3% vs 0.8% (*p* = 0.34). Major bleeding: 0% vs 1.4%.	Net clinical benefit favored stopping OAC, driven by bleeding reduction. Stroke equivalence not established; only 4 ischemic strokes across 840 patients.
OCEAN [5]	1284	Post-ablation, ≥1 yr stable, ≥1 stroke risk factor; multinational (154 sites, 18 countries)	≈2.2	Rivaroxaban 15 mg vs aspirin 70–120 mg	3 years planned; stopped early for futility (median ≈21 mo)	Protocol-specified arrhythmia-free status ≥1 yr pre-randomization; monitoring per local practice; brain MRI at baseline and 36 mo for covert stroke	Primary composite (stroke/SE/covert embolic infarct): RR 0.56; 95% CI 0.19–1.65; absolute diff at 3 yr −0.6 pp; *p* = 0.28. Fatal/major bleeding: HR 2.51; 95% CI 0.79–7.95. CRNM bleeding: ≈3.5× higher with rivaroxaban (*p* < 0.05).	Incremental embolic benefit of continued DOAC over aspirin was tiny; bleeding cost was real. Result reflects low ambient stroke risk post-ablation, not aspirin efficacy (aspirin has no proven stroke prevention in AF).

SE, systemic embolism; DOAC, direct oral anticoagulant; OAC, oral anticoagulation; CRNM, clinically relevant non-major; RR, relative risk; HR, hazard ratio; CI, confidence interval; mo, months; pp, percentage points; MRI, magnetic resonance imaging; AF, atrial fibrillation; ECG, electrocardiogram; yr, year. Note: Neither trial was designed or powered as a noninferiority study for stroke as an isolated endpoint; confidence intervals are wide and cannot exclude a 2- to 3-fold increase in stroke risk with de-escalation.

ALONE-AF asked the most direct question: in patients arrhythmia-free for ≥12 months beyond a blanking period, with CHA_2_DS_2_-VASc ≈2, can the direct oral anticoagulant (DOAC) be discontinued entirely? Ischemic stroke and systemic embolism (SE) were uncommon in both groups (2-year cumulative rates 0.3% vs 0.8%). The primary composite endpoint of stroke, SE, and major bleeding favored discontinuation (0.3% vs 2.2%; *p* = 0.02), largely reflecting fewer major bleeding events in the discontinuation arm [4].

OCEAN compared continued rivaroxaban with low-dose aspirin in a similar population (mean CHA_2_DS_2_-VASc 2.2) and found no significant difference in the primary composite of stroke, systemic embolism, or covert embolic infarct (0.31 vs 0.66 events per 100 patient-years; relative risk 0.56; 95% confidence interval(CI)0.19–1.65; *p* = 0.28). Bleeding was higher with rivaroxaban, with fatal or major bleeding occurring in 1.6% vs 0.6% (hazard ratio 2.51; 95% CI 0.79–7.95), and clinically relevant non-major bleeding was approximately 3.5-fold higher. The trial was stopped early for futility [5]. It is important to note that aspirin has not demonstrated stroke prevention efficacy in AF in prior trials (BAFTA, AVERROES) [6,7]; the OCEAN finding should therefore be interpreted as evidence of low post-ablation stroke risk in this cohort rather than as an endorsement of aspirin as a stroke prevention agent.

### Critical Limitations of the Evidence

Several features temper the strength of these findings. First, neither trial was designed or powered as a noninferiority study for stroke or systemic embolism as an isolated endpoint. Event rates were extremely low, producing wide confidence intervals that cannot exclude a 2- to 3-fold increase in stroke risk with de-escalation [4,5]. Second, follow-up was relatively short (2–3 years), whereas ablation freedom-from-AF curves decline progressively over 5+ years [8]. A strategy that appears safe at 2 years may not remain so at 5 or 10 years if AF recurs, and most authorities acknowledge that catheter ablation does not “cure” AF in many patients. Third, the enrolled populations were carefully selected and rigorously monitored under conditions that may not be replicated in routine clinical practice where surveillance intensity is typically lower. Finally, the trials predominantly enrolled patients with CHA_2_DS_2_-VASc ≈2; although approximately 30% of enrolled patients had scores of 3 or higher, the number of events in this subgroup was small, limiting the precision of risk estimates and warranting caution when extrapolating to higher-risk patients.

Additionally, the ALONE-AF primary composite has been criticized for merging the benefit of anticoagulation (stroke prevention) with its harm (bleeding) into a single endpoint, potentially conflating a reduction in treatment-related harm with therapeutic equivalence [9]. Emerging meta-analytic data pooling these trials with earlier observational studies suggest that OAC discontinuation may be reasonable when CHA_2_DS_2_-VASc is ≤2–3 but that the safety signal attenuates in higher-risk populations [10].

A more recent and substantially larger meta-analysis by Matteucci and colleagues pooled 28 studies comprising 267,443 post-ablation patients and found that OAC discontinuation markedly reduced the composite of thromboembolism and major bleeding (relative risk (RR) 0.44; 95% CI 0.32–0.61), driven by fewer bleeding events (RR 0.25; 95% CI 0.16–0.39), without a statistically significant excess of thromboembolism (RR 0.84; 95% CI 0.64–1.12) [11]. Findings were consistent across subgroups defined by study design, CHA_2_DS_2_-VASc score, and geography, although funnel-plot analysis raised the possibility of small-study effects for the bleeding outcome, and the confidence interval for thromboembolism cannot exclude a modest increase in stroke risk. Whether ablation energy source modifies post-ablation thromboembolic risk remains unresolved. These data lend weight to the view that OAC discontinuation is feasible in carefully selected patients, while leaving open whether it is equivalent to continued anticoagulation for stroke prevention.

## 4. Registries and Real-World Post-Ablation Risk

The randomized trials sit atop a larger body of observational data that points in a broadly consistent direction: post-ablation stroke risk is generally low, but safety of stopping OAC depends on baseline risk, structural phenotype, and timing. Noseworthy et al. [3] demonstrated in a large US claims dataset that stopping OAC within the first 3 months after ablation was associated with markedly higher cardioembolic events across all risk groups, whereas discontinuation beyond 3 months remained hazardous primarily in patients with CHA_2_DS_2_-VASc ≥2. Kanaoka et al. [12], analyzing over 230,000 post-ablation patients in a Japanese national database, found that OAC continuation conferred little thromboembolic benefit but a clear bleeding penalty in low-risk patients (CHADS_2_ ≤1), whereas those with CHADS_2_ ≥3 derived significant net benefit from continued anticoagulation. Fei et al. [13] reported similar findings in a Chinese cohort, with excess thromboembolic risk from OAC discontinuation concentrating in higher-risk patients. Iwawaki et al. [14] landmarked 1821 patients at 12 months of arrhythmia freedom and found that harm from stopping OAC clustered in patients with asymptomatic AF, reduced left ventricular ejection fraction, or enlarged left atrium, structural markers of more advanced atrial substrate.

Table 2 (Ref. [3,12,13,14]) summarizes these registries. Collectively, they reinforce universal OAC in the early post-ablation period, suggest that low-to-intermediate-risk patients with favorable substrate can often de-escalate safely, and identify high thromboembolic risk, adverse structural markers, and silent AF as features that make stopping OAC dangerous despite apparently successful ablation.

**Table 2.** **Key post-ablation registry data on OAC continuation versus discontinuation**.

Study [Ref]	N	Population / Design	Key Finding: OAC Discontinuation	Risk Modifiers Identified	Clinical Implication
Noseworthy et al. [3]	6886	US claims database (2005–2014); post-ablation AF patients; OAC patterns analyzed by timing of discontinuation	Stopping OAC within 3 mo: higher cardioembolic events across all risk groups. Beyond 3 mo: higher risk concentrated in CHA_2_DS_2_-VASc ≥2	Timing of discontinuation; baseline CHA_2_DS_2_-VASc score	Early post-ablation OAC essential universally; late discontinuation risky mainly in higher-risk patients. Supports a time-dependent approach to de-escalation decisions.
Kanaoka et al. [12]	>230,000	Japanese national database (2012–2019); post-ablation patients; used CHADS_2_ (not CHA_2_DS_2_-VASc)	Low-risk (CHADS_2_ ≤1): no thromboembolic benefit from OAC, increased bleeding. CHADS_2_ ≥3: net benefit from continued OAC	CHADS_2_ score threshold; bleeding risk	Risk-stratified approach justified; low-risk patients may safely de-escalate. Largest post-ablation dataset supporting differential benefit by baseline risk.
Fei et al. [13]	2117	Chinese cohort; single-center; post-ablation OAC discontinuation outcomes	Excess thromboembolic risk from discontinuation concentrated in higher-risk patients	Baseline thromboembolic risk	Confirms risk-dependent safety of discontinuation across ethnically diverse populations; consistent with Japanese and US data.
Iwawaki et al. [14]	1821	Japan; multicenter; patients landmarked at 12 mo arrhythmia freedom post-ablation	Harm from stopping OAC clustered in patients with asymptomatic AF recurrence, reduced LVEF, or enlarged LA	Asymptomatic AF; reduced LVEF; LA enlargement	Substrate markers identify at-risk patients even with apparent ablation success. Highlights that structural phenotype matters beyond rhythm status alone.

AF, atrial fibrillation; OAC, oral anticoagulation; LA, left atrial; LVEF, left ventricular ejection fraction; mo, months. Kanaoka et al. [12] used CHADS_2_ rather than CHA_2_DS_2_-VASc.

## 5. Why Post-Ablation CHA_2_DS_2_-VASc May Represent a Different Risk State

### 5.1 The Role of AF Burden

Understanding why post-ablation stroke risk appears lower than predicted requires clarity about AF burden as a concept. As articulated in the 2025 European Society of Cardiology (ESC)/European Heart Rhythm Association (EHRA) clinical consensus statement [15], AF burden is properly defined as the percentage of time spent in AF over a monitoring period, and should be distinguished from AF episode duration (the length of the longest single episode) and AF density (clustering of episodes). No validated threshold of AF burden below which stroke risk is negligible has been established [15], and this uncertainty is a fundamental limitation of any burden-based de-escalation strategy.

A chronological arc of device-based studies has progressively illuminated the relationship between atrial tachyarrhythmia burden and thromboembolic risk, while consistently failing to identify a safe lower threshold. The MOde Selection Trial (MOST) substudy by Glotzer and colleagues was the first to link device-detected atrial high-rate episodes to clinical outcomes: in 312 patients with sinus node dysfunction, any episode exceeding 5 minutes independently predicted death or nonfatal stroke (hazard ratio 2.79; 95% CI 1.51–5.15) [16]. TRENDS subsequently showed that device-detected atrial tachycardia ≥5.5 hours on any day roughly doubled thromboembolic risk, whereas shorter daily burdens conferred rates similar to patients without detected arrhythmia [17]. ASSERT found that subclinical AF lasting ≥6 minutes carried an approximately 2.5-fold higher stroke risk [18]. The heterogeneity of thresholds across these studies—5 minutes, 6 minutes, and 5.5 hours—underscores both the plausibility of a dose–response relationship between AF burden and stroke and the enduring absence of a clinically validated cutoff on which a de-escalation decision could be anchored. Together they support a model in which CHA_2_DS_2_-VASc captures the substrate for stroke and AF exposure adds a multiplicative risk modifier.

Catheter ablation does not change age or vascular biology, but in many patients it dramatically reduces AF exposure, sometimes to undetectable levels on continuous monitoring for years. In ALONE-AF and OCEAN, this low-AF state was defined pragmatically–typically ≥12 months without documented AF on scheduled monitoring–rather than by a specific burden threshold. If thromboembolic risk is a joint product of substrate and AF exposure, then a patient with CHA_2_DS_2_-VASc 2 and frequent, prolonged AF episodes is not equivalent to the same patient with CHA_2_DS_2_-VASc 2 and near-zero AF on an ILR 18 months after ablation. The numerical score is identical; we hypothesize that the effective risk is not. The low stroke rates observed in ALONE-AF and OCEAN are consistent with this hypothesis, falling well below the 2–3%/year historically ascribed to CHA_2_DS_2_-VASc 2–3 in non-ablated AF. A low group-level event rate does not, however, imply negligible individual risk; a residual stroke risk may remain clinically meaningful at the level of the individual patient even when absolute trial rates are small.

### 5.2 Atrial Cardiomyopathy and the Healthy-Responder Question

An important alternative interpretation must be acknowledged: patients who respond favorably to ablation and maintain sinus rhythm for 12+ months may have inherently less advanced atrial substrate than those who recur, and their low stroke rate may reflect this more benign phenotype rather than (or in addition to) a direct effect of AF elimination. The concept of atrial cardiomyopathy, as formalized in the 2024 EHRA/Heart Rhythm Society (HRS)/Asia Pacific Heart Rhythm Society (APHRS)/Latin American Heart Rhythm Society (LAHRS) consensus statement [19], encompasses structural, contractile, electrical, and fibrotic abnormalities of the atria that can exist independently of AF and that contribute to thromboembolism through stasis, endothelial dysfunction, and hypercoagulability. Patients with extensive atrial fibrosis, severely dilated left atria, or markedly impaired atrial mechanical function may remain at elevated stroke risk even if sinus rhythm is restored, while those with minimal fibrosis and preserved atrial function may have been at lower risk all along.

This healthy-responder bias does not invalidate the clinical observation that arrhythmia-free post-ablation patients have low stroke rates; rather, it reframes the mechanism. Whether ablation reduces risk by eliminating AF, or whether ablation success identifies patients whose atrial substrate was always more benign, or both, the practical implication converges: post-ablation risk stratification should incorporate atrial substrate assessment. We recommend operationalizing substrate assessment using specific metrics: left atrial (LA) volume index (normal <34 mL/m^2^ per American Society of Echocardiography (ASE) guidelines), left atrial reservoir strain (e.g., markedly impaired if <16%, though universally validated thresholds are lacking), left ventricular ejection fraction (LVEF), and where available, late gadolinium enhancement on cardiac magnetic resonance imaging (MRI) as a fibrosis surrogate. The staging system proposed in the 2024 atrial cardiomyopathy consensus [19] offers a structured vocabulary for this assessment. We acknowledge that, unlike CHA_2_DS_2_-VASc, these substrate metrics are not yet standardized into a simple bedside tool, and access to cardiac MRI for fibrosis assessment is limited in many practice settings. This represents a practical barrier to implementing substrate-guided risk stratification, and further work is needed to translate these concepts into clinically accessible instruments.

### 5.3 Monitoring Considerations

The validity of any de-escalation strategy hinges on the quality of rhythm surveillance used to define ablation “success”. A critical but often underappreciated point is that different monitoring modalities have vastly different sensitivity for detecting AF. Implantable loop recorders (ILRs) and cardiac implantable electronic devices (CIEDs) with atrial leads provide continuous monitoring and provide the most sensitive surveillance for excluding subclinical AF. Extended continuous patch monitors (14–30 days) offer reasonable but time-limited surveillance. Serial standard Holter monitors (24–48 hours) and intermittent electrocardiograms (ECGs) have substantially lower sensitivity and will miss the majority of brief, asymptomatic episodes [20].

In ALONE-AF and OCEAN, monitoring protocols were more rigorous than typical clinical practice: ALONE-AF required systematic rhythm surveillance before randomization, and OCEAN confirmed stability over ≥1 year. The low event rates in these trials may therefore partly reflect the rigor of screening, which excluded patients with undetected silent AF recurrence–a filter that may not be replicated in routine care where monitoring intensity is often lower. Clinicians considering OAC de-escalation should critically appraise whether their monitoring intensity approximates trial conditions; the lower the surveillance intensity relative to these trial standards, the greater the uncertainty about whether apparent arrhythmia freedom is genuine. We suggest a tiered approach: continuous monitoring via ILR or CIED is preferred; when unavailable, serial extended ambulatory monitoring (e.g., ≥14-day patch monitors every 6 months for at least 2 years) represents a reasonable minimum. Intermittent brief Holter or office ECG alone is insufficient to confidently exclude silent AF recurrence.

## 6. Applying the Evidence: A Domain-Based Framework

The data reviewed above do not establish that anticoagulation is obsolete after AF ablation; they generate a hypothesis—exploratory rather than practice-changing—that a score-only approach may be oversimplified once AF exposure has been durably lowered in well-characterized patients. We identify five domains across which clinical uncertainty about continued OAC varies: (1) time since ablation, (2) baseline thromboembolic risk, (3) rhythm monitoring quality, (4) AF burden, and (5) atrial substrate. Each has been discussed in the preceding sections. When all five domains are favorable, the question of whether OAC remains necessary can be raised with reference to trial data rather than extrapolation alone–but “can be raised” is not synonymous with “should be stopped”. When any domain is unfavorable, uncertainty increases and the case for continued OAC strengthens. No single domain is sufficient to mandate or prohibit de-escalation; the interaction among domains in any individual patient requires clinical judgment and shared decision-making.

Returning to the patient from our introduction–a 65-year-old man with paroxysmal AF and diabetes, arrhythmia-free on ILR at 18 months post-ablation, troubled by bruising on apixaban:

In this patient, all five domains are favorable. This is the clinical profile in which the question of OAC continuation can be discussed with reference to randomized data showing very low event rates. Such a conversation requires explicit acknowledgment of the residual uncertainties outlined earlier in this review, and a shared plan for ongoing rhythm surveillance with a low threshold to restart anticoagulation if AF recurs or risk factors evolve.

## 7. Research Agenda

If we accept that ablation may recalibrate–rather than erase–the stroke risk associated with a given CHA_2_DS_2_-VASc score, several evidence gaps must be addressed. First, prospective, AF burden–guided OAC trials in post-ablation patients with continuous monitoring are needed, randomizing after a defined period of low AF burden (≥12–24 months on ILR or CIED) to continued DOAC versus step-down strategies, with stroke, major bleeding, and cognitive outcomes as hierarchical endpoints. Second, integrated risk models combining the domains discussed above may discriminate post-ablation risk more precisely than any single domain alone. Third, the comparative effectiveness of different de-escalation strategies requires study in older, more comorbid populations representative of everyday practice. Fourth, long-term follow-up (≥5 years) of post-ablation cohorts is essential to determine whether early de-escalation strategies remain safe as late AF recurrence accumulates. Fifth, rhythm-triggered “pill in the pocket” anticoagulation–initiating OAC only when AF is detected on continuous monitoring [21]–is a promising concept that could individualize treatment more precisely than a binary continue-or-stop approach, though it requires prospective validation before adoption. Finally, factor XIa inhibitors, which substantially reduce bleeding but whose stroke prevention efficacy remains unproven after the early termination of OCEANIC-AF [22], may eventually alter the risk–benefit calculus of post-ablation anticoagulation if ongoing trials such as LIBREXIA AF [23] demonstrate a more favorable efficacy-to-safety profile.

## 8. Conclusion

The central hypothesis of this review is that a CHA_2_DS_2_-VASc score does not map to a fixed stroke risk once AF exposure has been durably reduced by ablation. The clinical substrate remains, but in appropriately selected post-ablation patients with low AF burden and without advanced atrial cardiomyopathy, the joint product of substrate and AF exposure may yield lower absolute stroke risk than historical estimates. ALONE-AF and OCEAN are consistent with this, showing that annualized stroke rates in such patients are extremely low and that the risk–benefit trade-off may shift toward bleeding avoidance.

The conversation about OAC de-escalation after ablation is no longer evidence-free, but it remains evidence-limited. Within the narrow phenotype studied in ALONE-AF and OCEAN–patients with 1–2 non-sex CHA_2_DS_2_-VASc risk factors who are arrhythmia-free beyond 12 months with robust monitoring and without advanced atrial cardiomyopathy, clinicians and patients can discuss the possibility of stopping OAC with reference to randomized data showing very low event rates, while honestly acknowledging that these trials were not designed to prove equivalence and that long-term safety is unknown. For the majority of post-ablation patients–particularly those with higher thromboembolic risk, advanced atrial substrate, or inadequate monitoring–guideline-directed OAC remains the standard. The path forward requires better evidence: longer follow-up, integrated risk models, and prospective burden-guided trials.

## Abbreviations

AF, atrial fibrillation; ASE, American Society of Echocardiography; CIED, cardiac implantable electronic device; DOAC, direct oral anticoagulant; ECG, electrocardiogram; ILR, implantable loop recorder; LA, left atrial; LVEF, left ventricular ejection fraction; MRI, magnetic resonance imaging; OAC, oral anticoagulation; SE, systemic embolism.
